# Cognitive Neuroscience of Autism Spectrum Disorder: The Neurobiology of Empathic Process

**DOI:** 10.1192/j.eurpsy.2024.1289

**Published:** 2024-08-27

**Authors:** C. Pinheiro Ramos, J. Marta, M. J. Freire, S. Mendes, A. Gamito

**Affiliations:** Setúbal Hospital Center, Setúbal, Portugal

## Abstract

**Introduction:**

Autism Spectrum Disorders (ASD) is diagnosed when an individual displays irregularity in three key domains: social development, communication, and repetitive behavior/obsessive interests.

The theory of mind-blindness in ASD suggests that individuals on the autism spectrum exhibit deficiencies in the typical empathic process, relative to their mental age.

Empathy comprises two primary components: firstly, the capacity to attribute mental states to both oneself and others, and secondly, experiencing an emotional response that aligns with the mental state of the other person.

**Objectives:**

This study aimed to synthetase the latest evidence about the neuropsychiatric basis of empathy in ASD.

**Methods:**

A review was conducted, drawing on reputable sources (PubMed and Web of Science databases).

**Results:**

A neural basis of empathy has built on a model first proposed by Brothers. It was suggested that social intelligence was a function of three regions: the amygdala, the orbitofrontal and medial frontal cortex, and the superior temporal sulcus and gyrus - the “social brain”. Abnormalities in autism have been found in the amygdala, the orbito and the medial frontal cortex.

Amygdala has been implicated primarily in fear perception of facial expressions, as well as in the recognition of other emotions such as sadness and “social” emotions. In addition to fear perception, the amygdala has also been implicated in related processes including eye gaze, affective memory, olfactory learning, and social judgment.

To date, findings on amygdala structure in autism have been mixed, with studies indicating reduced and increased volumes, as well as nonsignificant differences.

**Conclusions:**

ASD is one of the most heterogeneous neurodevelopmental disorders, and cognitive theories as well as structural findings have linked likely frontal lobe abnormalities to the social and cognitive profiles of autism.

Future studies may elucidate existing data by taking advantage of new and infrequently used data acquisition technologies such as Transcranial Magnetic Stimulation.

**Disclosure of Interest:**

None Declared

